# Heterologous Expression of ZmAHL10 Gene Enhances Low Nitrogen Tolerance in Transgenic Arabidopsis

**DOI:** 10.3390/plants15071062

**Published:** 2026-03-31

**Authors:** Junfei Liu, Yan Li, Guanqiang Zuo, Jinchong Li, Hao Shi, Shiwen Wang

**Affiliations:** 1 State Key Laboratory of Soil and Water Conservation and Desertification Control, The Research Center of Soil and Water Conservation and Ecological Environment, Chinese Academy of Sciences, Ministry of Education, Yangling, Xianyang 712100, China; liujunfei1021@163.com (J.L.);; 2 Institute of Soil and Water Conservation, Chinese Academy of Sciences, Ministry of Water Resources, Yangling, Xianyang 712100, China; 3 University of Chinese Academy of Sciences, Beijing 100049, China; 4 College of Natural Resources and Environment, Northwest A&F University, Yangling, Xianyang 712100, China; 5 College of Soil and Water Conservation Science and Engineering, Northwest A&F University, Yangling, Xianyang 712100, China

**Keywords:** AHLs, low nitrogen stress, Maize (*Zea mays* L.), nitrogen uptake, chlorophyll

## Abstract

Nitrogen is an essential element for plant growth, and low nitrogen stress significantly restricts crop yield. Therefore, cultivating crop varieties that are tolerant to low nitrogen is crucial for agricultural production. The AT-hook motif nuclear localization protein (*AHL*) family is vital for plant stress resistance. To investigate the potential regulatory mechanisms of the *AHL* family in maize under low nitrogen stress, 35 *ZmAHL* genes were identified from the maize genome using bioinformatics methods. The results indicated that these genes encode proteins with lengths ranging from 203 to 573 amino acids, with relative molecular weights between 20.68 and 59.68 kDa, and they are unevenly distributed across 10 chromosomes. Most proteins encoded by these genes are alkaline hydrophilic proteins, primarily localized in the nucleus. Family expansion occurred through tandem and fragment repeats, which exhibited evolutionary conservation with rice homologous genes. Transcriptome analysis revealed that the majority of *ZmAHL* genes in drought-tolerant maize inbred lines were significantly up-regulated under drought and low nitrogen stress, with the *ZmAHL10* gene displaying the most pronounced response to low nitrogen conditions. Experiments involving transgenic *Arabidopsis thaliana* further confirmed that the growth status, nitrogen uptake, and photosynthetic pigment content of *ZmAHL10* overexpression strains under low nitrogen conditions were superior to those of the wild type, while the mutant exhibited significant growth inhibition. Overall, this study delineated the fundamental characteristics of the maize *ZmAHL* gene family and established that *ZmAHL10* enhances low nitrogen tolerance in plants by improving nitrogen absorption capacity and maintaining the stability of the photosynthetic system. This research provides candidate genes and a theoretical foundation for the molecular breeding of maize with enhanced low nitrogen tolerance.

## 1. Introduce

Maize (*Zea mays* L.), recognized as the most widely cultivated and highest-yielding food crop globally [1], serves as a fundamental crop for ensuring food security. It is characterized by high nitrogen requirements and moderate drought tolerance [2]. As the application of maize expands into bioenergy, feed processing, and other industries, the market and industrial demands for its yield and quality have steadily increased. Nevertheless, abiotic stresses, including low nitrogen availability and drought, significantly hinder both yield formation and quality enhancement in maize [3]. Recently, investigating the mechanisms underlying crop responses to low nitrogen stress has emerged as a focal point in the field of molecular breeding for stress resistance [4]. This research provides crucial genetic resources and theoretical support for the development of low nitrogen-tolerant crop varieties and the enhancement of nitrogen fertilizer utilization efficiency.

Nitrogen is a vital macronutrient for plant growth and development, constituting a fundamental component of biological macromolecules such as proteins, nucleic acids, and chlorophyll. It is also a critical factor that limits crop yield and quality [5,6]. Approximately 40% of global grain production depends on the application of chemical nitrogen fertilizers; however, excessive use leads not only to resource wastage but also to significant environmental issues, including soil acidification and water eutrophication [7,8]. Concurrently, global climate change has heightened the frequency of various abiotic stresses, such as drought, which interacts with low nitrogen levels to create a combined-stress environment that inflicts compounded damage on crop production [9]. In light of the pressures posed by population growth, ecological preservation, and climate anomalies, it is essential to cultivate crop varieties that are tolerant to low nitrogen conditions and to analyze the molecular regulatory networks that govern plant responses to low nitrogen stress. This approach is crucial for enhancing nitrogen fertilizer use efficiency and ensuring the stability of food production [10].

Nitrogen is a limiting nutrient element for plant growth and development, and its insufficient supply will lead to a series of physiological, biochemical and molecular changes in plants, including nitrogen absorption, transport and assimilation [11]. On the one hand, plants expand the absorption area by adjusting root morphology (such as increasing lateral root density and prolonging the length of main root) [12]. Meanwhile, the activities of nitrogen transporters (e.g., NRT1 and NRT2 families) and key nitrogen assimilation enzymes (e.g., nitrate reductase (NR) and glutamine synthetase (GS)) are enhanced to improve the absorption and utilization efficiency of limited nitrogen [13,14]. In addition, low nitrogen stress can also lead to the reconstruction of carbon and nitrogen metabolism balance in plants, the adaptive adjustment of the photosynthetic system, and the activation of the antioxidant system, so as to reduce energy consumption and alleviate oxidative damage [15]. At the molecular level, nitrogen signals are sensed by receptor proteins, which activate regulatory networks including myb, NAC, bZIP and other transcription factor families and synergistically regulate the spatio-temporal expression of genes related to nitrogen metabolism [16,17,18]. As the core node of the regulatory network, transcription factors regulate the expression of downstream genes by specifically binding to the promoter region of target genes and play a key role in plant stress adaptation [19]. Among these, the AT-hook motif nuclear localization protein (AHL) family, as a class of conserved transcriptional regulators, has gradually become a research focus in plant stress resistance. This is because *AHLs* contain AT-hook domains that specifically bind to AT-rich regions of DNA and DUF296 domains that participate in protein–protein interactions [20].

The evolutionary conservation and functional diversity of *AHL* family genes have been confirmed in many plants, which not only participate in the regulation of plant growth and development but also play an important role in abiotic stress response [21]. Existing studies have shown that the *AHL* gene family plays a key regulatory role in plant growth and development and drought stress response. In *Arabidopsis thaliana*, this family contains 29 members. The members of this family have diverse functions in plants, being able to regulate the growth processes of hypocotyl elongation and fruit development and also mediate the signal response to abiotic stresses such as drought, high temperature and salt stress [22,23]. For example, *Arabidopsis atahl10* mainly negatively regulates plant growth under drought stress and enhances drought adaptability by inhibiting growth. Its effect depends on ser314 phosphorylation, which mediates chromatin recruitment of the epigenetic factor rrp6l1 and cooperatively inhibits the expression of growth-related genes [24]. In other species, such as rice *osahl1*, drought resistance can be enhanced [25]. In summary, AHL integrates developmental and environmental signals and is an important candidate gene for crop genetic improvement.

Previous studies have confirmed that significant differences in low nitrogen and drought tolerance exist among different maize varieties, and these differences are closely related to the expression patterns of stress-responsive genes [26]. Nevertheless, the regulatory mechanism of the *AHL* gene family in maize remains largely unclear [27]. The core objective of this study is to systematically analyze the basic characteristics of the *ZmAHL* gene family in maize, identify key genes involved in low nitrogen stress response within this family, and clarify their mechanisms of regulating maize low nitrogen tolerance. Initially, this study utilized bioinformatics methods to identify members of the *ZmAHL* gene family from the maize genome and systematically analyzed their physicochemical characteristics, gene structure, chromosomal distribution, and other features. Combined with RNA-seq analysis, the core *ZmAHL10* gene involved in maize’s response to low nitrogen stress was screened. Through heterologous expression experiments in *Arabidopsis thaliana*, the regulatory mechanism of the *ZmAHL10* gene through nitrogen accumulation and stabilizing the photosynthetic system was further elucidated.

## 2. Results

### 2.1. Identification of Zmahl Gene Family

To identify all *AHL* genes in maize, the hidden Markov model (HMM) of AHL (PF03479) was used as a query, leading to the identification of 35 *ZmAHL* genes in maize (see Supplementary Table S1). Bioinformatics analysis revealed the protein characteristics of this gene family: the amino acid length ranged from 203 residues (transcript: Zm00001eb339520) to 573 residues (transcript: Zm00001eb404380), with an average of 332.9 amino acids. The corresponding molecular weight varied from 20.68 kDa to 59.68 kDa (average: 33.77 kDa). The predicted isoelectric point ranged from 5.08 (transcript:zm00001eb381980) to 11.71 (transcript:zm00001eb390500), with an average of 8.06. Among them, 20 genes encode basic protein (pi > 7), 15 genes encode acidic protein (pi < 7), and 2 genes encode near neutral protein (PI ≈ 7), showing the characteristics of alkaline protein as a whole. Protein stability analysis showed that the instability index ranged from 32.42 to 78.89 (average: 57.36); only two genes encoded stable proteins (instability index < 40), while 33 genes encoded unstable proteins (instability index > 40). The aliphatic index ranged from 55.77 to 88.03 (average: 69.02), suggesting good thermal stability of the family proteins. Hydrophilicity analysis indicated that the average hydrophilicity (GRAVY) ranged from −0.540 to 0.263 (average: −0.238); 33 genes encoded hydrophilic proteins (GRAVY < 0), and only 2 genes encoded hydrophobic proteins (GRAVY > 0), indicating that ZmAHL proteins are mainly hydrophilic.

Subcellular localization prediction showed diverse distribution of ZmAHL proteins: they were mainly localized in the nucleus (32) and cytoplasm (25), followed by mitochondria (21), vacuoles (19), and chloroplasts (18); a small number were localized in the endoplasmic reticulum (12), Golgi apparatus (8), and peroxisomes (7). This extensive subcellular distribution provides a structural basis for the versatility of ZmAHL proteins.

### 2.2. Structural Characteristics of ZmAHL Gene Family

**Figure 1:**
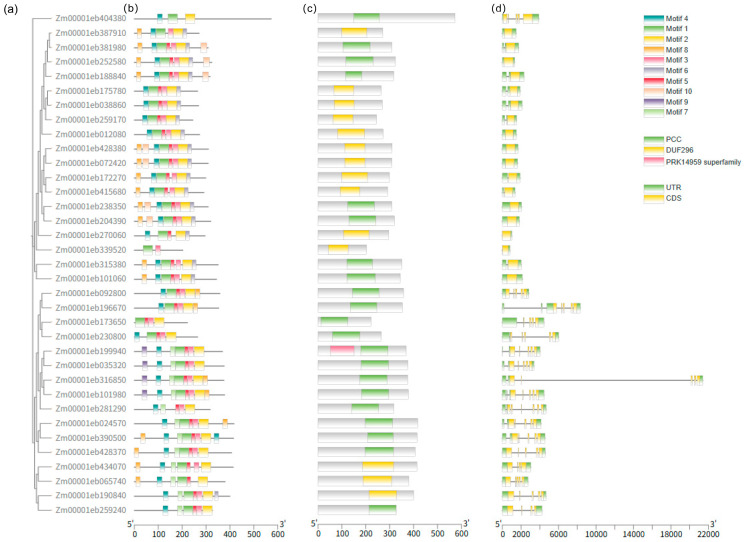
Analysis of protein structure and gene characteristics of maize zmahl gene family. (**a**) Phylogenetic tree analysis of MAHL family. (**b**) The conserved motifs of zmahl family proteins are distributed, and different colors represent different motifs. (**c**) Protein conserved domain analysis. (**d**) Exon–intron structure analysis showed the distribution of UTR (green box), exon (yellow box) and intron (black line) at the 5′ and 3′ ends of zmahl family genes.

In this study, the structural characteristics, conserved protein domains, and exon–intron structures of the maize *ZmAHL* gene family were systematically analyzed. Phylogenetic tree analysis (Figure 1a) revealed that ZmAHL family proteins can be divided into six subgroups. Ten conserved protein motifs were identified via MEME motif analysis (Figure 1b). All ZmAHL family proteins contain these 10 conserved motifs, among which motifs 1, 2, and 3 are shared by all members, forming the core functional region of the family. The distribution pattern of these motifs also reflects the evolutionary conservation and specificity of family members, providing important clues for subsequent functional verification. Conserved protein domain analysis (Figure 1c) confirmed that all zmahl family proteins contained highly conserved AT-hook domains and duf296 domains. The AT-hook domain is responsible for binding to the at-rich region of DNA, while the duf296 domain may be involved in protein interaction. The two domains together constitute the core functional module of this family of proteins. Exon–intron structure analysis (Figure 1d) showed that the number of introns in *ZmAHL* family genes ranges from 0 to 6; most contain 2–3 introns, and genes within the same subgroup exhibit high similarity in their structures. This structural conservation reflects the evolutionary relatedness of family members and also provides a structural basis for the conservation of gene functions.

### 2.3. Distribution of Zmahl Gene Family on Chromosome

**Figure 2:**
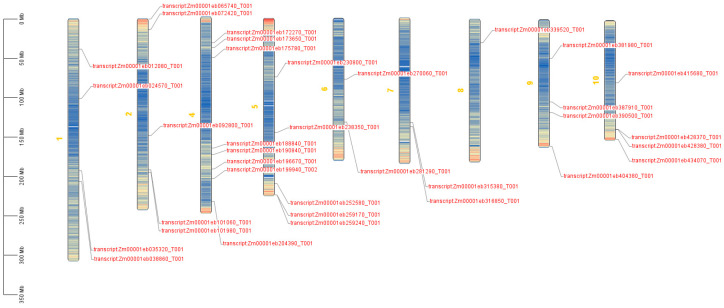
Chromosome distribution of members of zmahl gene family in maize. Color bands represent gene density. The vertical axis is the physical length of chromosome (MB). Chromosome numbers are marked on the left side of each figure.

To analyze the evolutionary expansion characteristics of the *ZmAHL* family genes, we investigated the localization of *ZmAHL* family genes on 10 maize chromosomes. The results showed that *ZmAHL* family genes were unevenly distributed on chromosomes with obvious clustering characteristics (Figure 2): chromosomes 1, 2, 4, 5, 9, and 10 were gene-dense distribution regions. Among these, the long arm region of chromosome 4 formed the densest gene cluster of this family, including members such as Zm00001eb101980 and Zm00001eb161580, suggesting that significant tandem duplication events occurred in this region. Obvious gene clustering regions were also observed on chromosomes 2, 5, and 10, whereas only 1–2 family members were present on chromosomes 6, 7, and 8. This non-random distribution pattern reflects the functional differentiation trend of the ZmAHL family, and the clustered genes may coordinately regulate the growth, development, or stress response processes of maize.

### 2.4. Intraspecific and Interspecific Collinearity Analysis of Zmahl Gene Family

**Figure 3:**
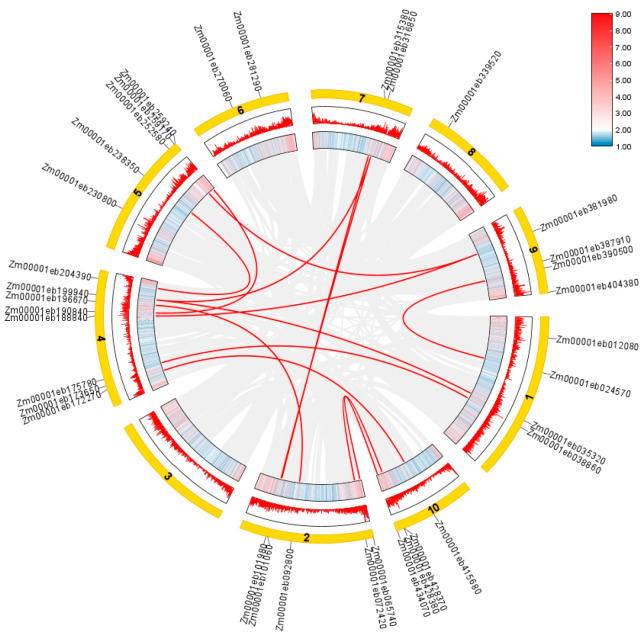
Intraspecific homolinearity analysis ring chart of maize ZmAHL family genes. Tbtools were used to analyze the homolinearity of *ZmAHL* family genes in maize chromosomes. The inner ring block was the physical map of chromosomes, the red block marked the dense distribution of genes, the red line represented significant homolinearity of gene pairs, and the yellow outer ring was the chromosome number.

This study revealed the amplification and evolutionary mechanism of ZmAHL family genes in the maize genome through intraspecific collinearity analysis. The results of the ring chart (Figure 3) showed that the collinear blocks of *ZmAHL* family genes were mainly concentrated in specific segments of chromosomes 2, 4, and 5, and these segments highly overlapped with the gene-dense regions on the chromosomes, suggesting that tandem duplication is one of the core ways of family expansion. The red lines in the figure represent gene pairs with significant collinear relationships; short-distance lines within the same chromosome (e.g., the dense lines on chromosome 4) confirm the high frequency of tandem duplication events, while long-distance connections across chromosomes (e.g., the association between chromosomes 2 and 7) reflect the contribution of segmental duplication to family expansion. These results indicate that the *ZmAHL* family can expand through the synergistic effect of tandem duplication and segmental duplication, providing direct genomic evidence for analyzing the evolutionary dynamics and functional differentiation of this family.

**Figure 4:**
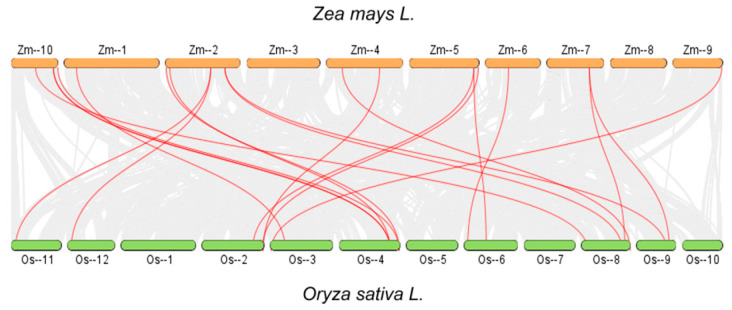
Species with the same linear relationship between maize and rice AHL family genes. The orange block represents the chromosome structure of maize, and the green block represents the chromosome structure of rice. The gray line in the background represents the whole-genome collinear blocks in the genomes of the two species, while the red line specifically highlights the collinear AHL gene pairs between maize and rice.

Interspecific collinearity analysis between maize (*Zea mays* L.) and rice (*Oryza sativa* L.) (Figure 4) revealed the cross-species evolutionary conservation and expansion characteristics of *ZmAHL* family genes. The results showed that maize *ZmAHL* family genes and their homologous genes in rice exhibited extensive collinear associations (red lines), and these associations displayed obvious chromosome preference: *ZmAHL* genes on maize chromosomes 2, 5, and 10 had the most collinear links with their homologous genes on rice chromosomes 2, 4, and 8, respectively. This reflects the high conservation of these chromosome segments following the differentiation of gramineous species. At the same time, some maize *ZmAHL* genes have multiple homologous copies in the rice genome, suggesting that the family experienced additional gene expansion events in the process of maize speciation. These findings indicate that *ZmAHL* gene not only has a wide range of pedigree-specific replication events but also has a conservative evolutionary relationship, especially in monocotyledonous plants.

### 2.5. Expression Pattern Analysis of Zmahl Gene Family Under Stress

**Figure 5:**
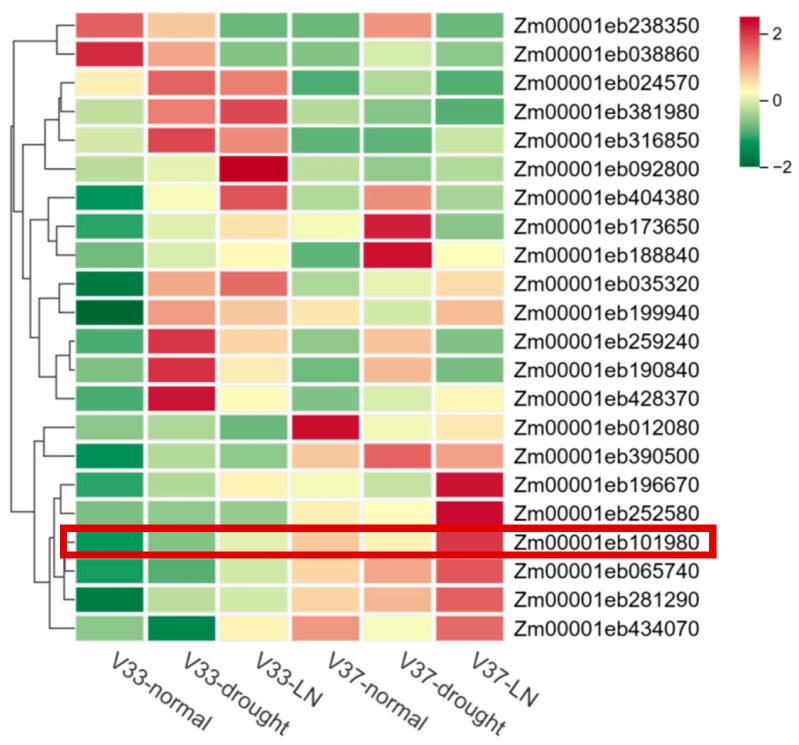
Hierarchical clustering heat map of differentially expressed genes in maize under drought and low nitrogen stress. The heat map shows the hierarchical clustering results of 20 key differentially expressed genes (DEG) between drought-tolerant variety V33 and non-drought-tolerant variety v37 under normal, drought and low nitrogen stress. The color gradient from green to red (corresponding to Z-score of −2 to 2) represents the relative expression level of the gene; The expression pattern of the ZmAHL10 gene is indicated within the red box, red indicates up-regulation and green indicates down-regulation.

Transcriptome results from the previous research group showed that the expression patterns of AHL genes differed significantly between drought-tolerant maize varieties V33 and V37 (Figure 5, Supplementary Table S2), with variety-specific responses observed: most *ZmAHL* genes in V33 were significantly up-regulated under drought and low nitrogen stress (red region), such as Zm00001eb173640 and Zm00001eb259240; in contrast, the expression of these genes in V37 showed no significant change or was down-regulated (green region). Regarding differences among stress types, the up-regulation of *ZmAHL* gene expression in V33 was greater under combined low nitrogen stress than under single drought stress, reflecting the synergistic response characteristics of this family to the two stresses. These results indicate that the differential expression of *ZmAHL* family genes is an important molecular basis for the drought resistance and low nitrogen tolerance of V33, providing key targets for subsequent functional verification.

**Figure 6:**
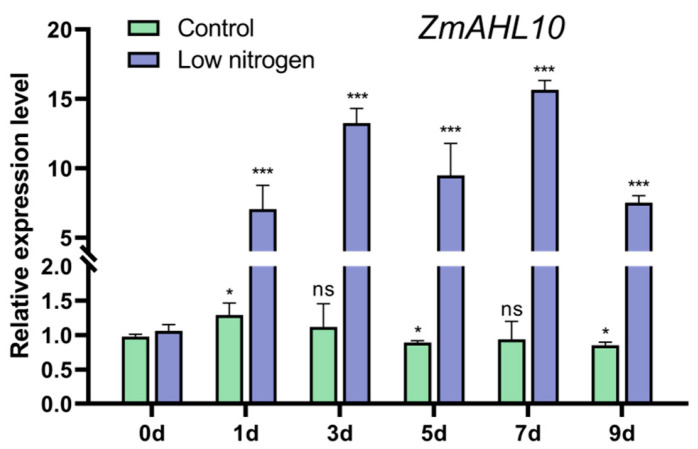
Expression levels of ZmAHL10 under low nitrogen treatment at different times. Maize inbred line B73 at the stage of five leaves and one core was treated with low nitrogen hydroponics (0.02 mm NH4NO3), and the leaves were taken for detection on days 0, 1, 3, 5, 7 and 9 after treatment. The histogram represents the mean ± standard deviation (*n* = 3) compared with 0 d under the same treatment. *: *p* < 0.05, ***: *p* < 0.001, NS: there is no significant difference using Student’s t test.

Among all *ZmAHL* genes, the expression of Zm00001eb101980 differed significantly between the two maize varieties. In both extreme varieties, Zm00001eb101980 expression was significantly down-regulated under drought treatment but up-regulated under low nitrogen treatment. Zm00001eb101980 showed the highest homology with Arabidopsis AHL10, so it was named *ZmAHL10*. Quantitative real-time PCR (qRT-PCR) was performed to detect the expression level of *ZmAHL10* at different time points under low nitrogen treatment (Figure 6), further indicating that it was significantly induced by low nitrogen stress.

### 2.6. Sequence Analysis and Subcellular Localization of ZmAHL10 Gene

**Figure 7:**
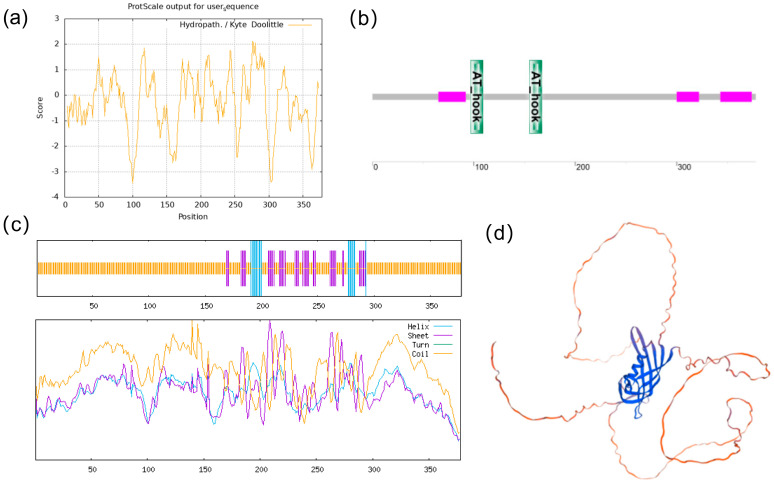
Sequence analysis of ZmAHL10 gene. (**a**) The hydrophobicity index curve of amino acid residues predicted by proscale module; negative value represents hydrophilic amino acid, and positive value represents hydrophobic amino acid. (**b**) ZmAHL10 protein functional domain distribution diagram; green box for the two at-hook domains, and pink box for the low-complexity region. (**c**) The secondary structure proportion curve predicted by ZmAHL10 protein. (**d**) The predicted three-dimensional structure model of ZmAHL10 protein.

**Figure 8:**
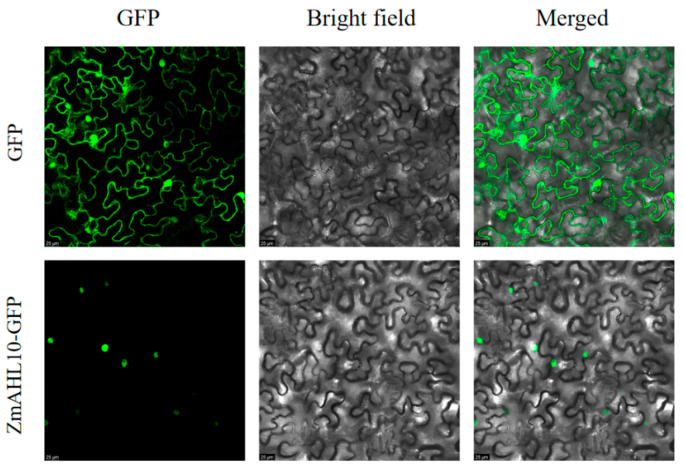
Subcellular localization analysis of ZmAHL10 protein. The fluorescence signal of *ZmAHL10* GFP group was limited to the nuclear region. The bright field image was used to display the cell contour, and the merged image further verified the nuclear localization feature of *ZmAHL10*; the scale bar is set to 25 μm.

The full-length CDs of *ZmAHL10* are 1134 bp, encoding 378 amino acids (Table S1). The molecular formula of the protein is c1616h2586n478o512s9, and the molecular weight of the protein is about 37.19 kDa. Through the prediction and analysis of the hydrophobicity index of amino acid residues of ZmAHL10 protein by proscale software, it can be seen that the coverage of hydrophilic amino acids (hydrophobicity index < 0) in the sequence is significantly higher than that of hydrophobic amino acids (hydrophobicity index > 0), which can preliminarily infer that the protein is a hydrophilic protein (Figure 7a). The ZmAHL10 protein contains two AT-hook domains at amino acid positions 97–109 and 155–167 (Figure 7b). Analysis of the protein secondary structure (Figure 7c) showed that it comprises 313 random coils (82.80%), accounting for the largest proportion; 47 extended strands (12.43%); and 18 α-helical structural elements (4.76%). Subcellular localization experiments of ZmAHL10 demonstrated that it is localized in the nucleus (Figure 8), indicating that ZmAHL10 protein was a nuclear localization protein, suggesting that it mainly played its biological function by binding DNA in the nucleus and regulating the expression of downstream genes.

### 2.7. ZmAHL10 Enhanced the Tolerance of Transgenic Arabidopsis to Low Nitrogen Stress

**Figure 9:**
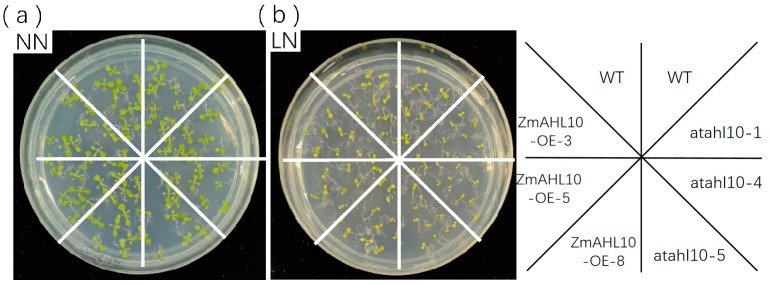
Growth phenotype of *Arabidopsis thaliana* seedlings under normal nitrogen (NN, (**a**)) and low nitrogen (LN, (**b**)) conditions of wild type (WT), *ZmAHL10* overexpression lines (-oe #3, -oe #5, -oe #8) and mutants (atah10-#1, atah10-#4, atah10-#5), with a diameter of 9 cm.

In view of the significant up-regulation of *ZmAHL10* expression under low nitrogen stress, we constructed *ZmAHL10* overexpression transgenic *Arabidopsis thaliana* strains and screened three homozygous T3 lines (OE#3, OE#5, and OE#8). Meanwhile, we obtained homozygous mutants (athl10#1, athl10#4, and athl10#5) of ATHL10—the Arabidopsis homologous gene of *ZmAHL10*—from an Arabidopsis mutant library. Transgenic plants and mutants were subjected to low nitrogen treatment and subsequent analysis. Under control conditions (modified Hoagland nutrient medium, 2 mM NH_4_NO_3_), no significant differences were observed among the wild type (WT, Col-0), overexpression transgenic lines, and mutant plants (Figure 9a). However, under low nitrogen stress (0.2 mM NH_4_NO_3_), the leaves of mutant lines exhibited significant growth retardation, whereas the leaf size of transgenic lines showed a phenotype similar to or slightly larger than that of the wild type (Figure 9b). The results showed that *ZmAHL10* could enhance the tolerance of plants to a low nitrogen environment and had little change on plant morphology under non-stress conditions, which supported its potential as a low nitrogen-tolerant variety breed.

### 2.8. ZmAHL10 Enhances Nitrogen Uptake by Transgenic Plants

**Figure 10:**
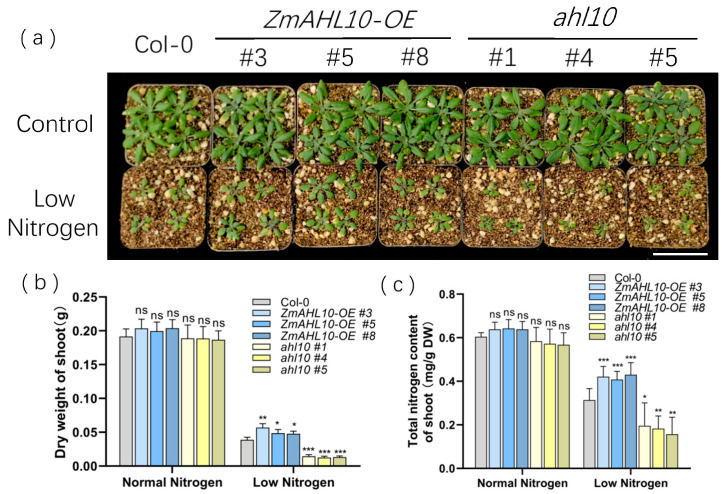
Overexpression of ZmAHL10 enhanced the tolerance of Arabidopsis thaliana to low nitrogen stress. (**a**) Phenotype comparison of *Arabidopsis thaliana* seedlings (Col-0, ZmAHL10 OE lines #3/#5/#8, and ahl10 mutant lines #1/#4/#5) under control (NN) and low nitrogen (LN) conditions; the scale bar is set to 5 cm. (**b**) Total nitrogen content in shoots of different strains under normal nitrogen and LN conditions. (**c**) The shoot dry weight of different strains under NN and LN conditions. The histogram represents the mean ± standard deviation (*n* = 3), and the asterisk indicates that there are statistically significant differences between other strains of plants and the wild type. Using Student’s t test, compared with Col-0 under the same treatment, *: *p* < 0.05, **: *p* < 0.01, ***: *p* < 0.001, ns: there is no significant difference.

To further verify the low nitrogen response function of *ZmAHL10*, we cultured wild type (WT, Col-0), overexpression lines (OE#3, OE#5, OE#8), and mutants (athl10#1, athl10#4, athl10#5) in soil under normal nitrogen and low nitrogen conditions, respectively, and observed them until 3 weeks old. The results showed that no phenotypic differences were observed among different genotypes when irrigated with a nutrient solution of normal nitrogen content. However, under low nitrogen stress, the overexpression lines exhibited significantly better leaf greenness and growth vigor than the wild type, whereas the knockout mutants showed obvious yellowing and growth inhibition (Figure 10a). Physiological index detection revealed that under low nitrogen treatment, the shoot dry weight of overexpression lines was significantly higher than that of the wild type, while the shoot dry weight of mutants was significantly lower than that of the wild type (Figure 10b). Meanwhile, consistent with the biomass results, the total nitrogen content of overexpression lines was significantly higher than that of the wild type under low nitrogen treatment, whereas the opposite was true for the mutants (Figure 10c). These results confirmed that *ZmAHL10* can enhance plant low nitrogen tolerance by promoting nitrogen absorption.

**Figure 11:**
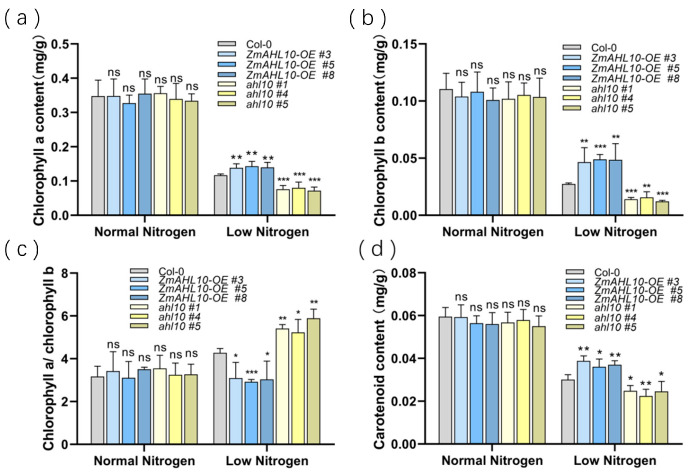
Analysis of photosynthetic pigment content of different strains of Arabidopsis thaliana under normal nitrogen and low nitrogen conditions. (**a**) Chlorophyll a content; (**b**) chlorophyll b content. (**c**) The ratio of chlorophyll a to chlorophyll b. (**d**) Carotenoid content. The histogram represents the mean ± standard deviation (*n* = 3), and the asterisk indicates that there are statistically significant differences between other strains of plants and the wild type. Using Student’s t test, compared with Col-0 under the same treatment, *: *p* < 0.05, **: *p* < 0.01, ***: *p* < 0.001; ns: there is no significant difference.

In addition, the chlorophyll content of *Arabidopsis thaliana* under different treatments was detected to evaluate the effect of *ZmAHL10* on the stability of the photosynthetic system. Under low nitrogen stress, the contents of chlorophyll a (Figure 11a) and chlorophyll b (Figure 11b) in overexpression lines were significantly higher than those in the wild type and mutants. The change trend of carotenoid content (Figure 11d) was consistent with that of chlorophyll. Notably, the chlorophyll a/b ratio usually increases under low nitrogen stress [28]. Under low nitrogen stress, the chlorophyll a/b ratio of ZmAHL10-OE *Arabidopsis thaliana* was significantly lower than that of the wild type, whereas the ratio in mutant lines was significantly higher (Figure 11c). These results indicated that overexpression of *ZmAHL10* can significantly alleviate the degradation of photosynthetic pigments caused by low nitrogen stress, enhance the stability of the photosynthetic system, and thereby improve plant low nitrogen tolerance.

## 3. Discussion

Plants have evolved a sophisticated molecular regulatory network to adapt to low nitrogen stress, in which transcription factors act as core regulatory nodes to precisely modulate the spatiotemporal expression of downstream stress-responsive genes, thus conferring plants the ability to cope with nitrogen deficiency [29,30]. The AT-hook motif nuclear localized (AHL) protein family is a class of highly conserved transcriptional regulators in plants, which harbors two characteristic functional domains: the AT-hook domain that specifically binds to AT-rich regions of genomic DNA, and the DUF296 domain that mediates protein–protein interactions [20,31]. Accumulating evidence has demonstrated that AHL family members are involved in the regulation of multiple plant biological processes, including growth and development as well as responses to various abiotic stresses such as drought, high salt and high temperature [21,22]. For instance, Arabidopsis AHL10 modulates plant growth under drought stress through phosphorylation-mediated chromatin recruitment of epigenetic factors [24], and rice *OsAHL1* enhances drought tolerance by regulating stress-related signal pathways [25]. However, the functional characterization of the AHL gene family in maize, a major cereal crop with high nitrogen demand, remains largely unexplored, especially its regulatory role and molecular mechanism in response to low nitrogen stress [27].

In this study, we systematically identified 35 *ZmAHL* genes from the maize B73 genome using a hidden Markov model (HMM) based on the conserved AHL domain (PF03479), which is more than the number of AHL members in Arabidopsis (29) [32] but less than that in polyploid crops such as Brassica napus [33]. This difference in gene number suggests that the *ZmAHL* family has undergone species-specific expansion during maize evolution, which may be an adaptive evolutionary strategy for maize to meet its high nitrogen demand and adapt to complex field stress environments [34]. Bioinformatics analysis revealed that ZmAHL proteins are predominantly alkaline hydrophilic proteins with an average isoelectric point of 8.06, and 33 members have a grand average of hydropathicity (GRAVY) value less than 0, which is a typical structural feature of nuclear-localized transcriptional regulators [30]. Meanwhile, the ZmAHL family has an average aliphatic index of 69.02, indicating good thermal stability of these proteins, which ensures their normal functional exertion under environmental temperature fluctuations. Subcellular localization prediction showed that 32 ZmAHL members are localized in the nucleus, which is consistent with the subcellular distribution characteristics of AHLs in wheat [35] and further confirms their role as transcription factors in regulating gene expression.

Phylogenetic and structural analysis further revealed the evolutionary conservation and functional specificity of the *ZmAHL* family. Phylogenetic tree construction divided the *ZmAHL* family into six distinct subgroups, and members within the same subgroup exhibited highly similar exon–intron structures and conserved motif distributions. MEME motif analysis identified 10 conserved motifs in the ZmAHL family, among which motifs 1, 2 and 3 are shared by all members, forming the core functional region of the family. This structural conservation is consistent with the characteristics of the AHL gene family in soybean and rice [36,37], which confirms the high evolutionary conservation of the AHL family in plants. Conserved domain analysis verified that all ZmAHL proteins contain both AT-hook and DUF296 domains, the two core functional domains of the AHL family [31]. The AT-hook domain enables ZmAHL proteins to bind to the AT-rich regions of target gene promoters, while the DUF296 domain mediates protein–protein interactions, and the cooperation of these two domains provides a structural basis for ZmAHL proteins to regulate downstream gene expression. Chromosome distribution analysis showed that *ZmAHL* genes are unevenly distributed on maize chromosomes with obvious clustering characteristics, and the long arm of chromosome 4 forms the densest gene cluster [38]. Interspecific collinearity analysis between maize and rice showed that *ZmAHL* genes on maize chromosomes 2, 5 and 10 have extensive collinear relationships with rice AHL homologous genes, reflecting the high conservation of AHL genes in gramineous plants. This evolutionary conservation provides a reliable basis for cross-species functional prediction of *ZmAHL* genes and lays the foundation for the study of AHL gene function in other gramineous crops.

Gene expression patterns under stress conditions are important clues for exploring gene functions [39,40]. In this study, transcriptome analysis of two maize varieties with different stress tolerances revealed significant variety-specific expression differences in *ZmAHL* genes under drought and low nitrogen stress. Most *ZmAHL* genes in the drought-tolerant variety V33 were significantly up-regulated under both drought and low nitrogen stress, and the up-regulation amplitude under low nitrogen stress was higher than that under single drought stress, while the expression of these genes in the non-drought-tolerant variety V37 showed no significant change or even down-regulation. This differential expression pattern is consistent with the general regulatory mechanism of stress-tolerant maize varieties, which efficiently activate the expression of stress-responsive genes to cope with environmental stresses [40], indicating that the differential expression of *ZmAHL* genes is an important molecular basis for the differences in low nitrogen and drought tolerance among maize varieties. Among all *ZmAHL* genes, *ZmAHL10* exhibits unique response characteristics. *ZmAHL10* has the highest homology with Arabidopsis AHL10, which is mainly involved in the regulation of drought stress response in Arabidopsis [24]. This functional divergence between *ZmAHL10* and its Arabidopsis homologous gene reflects the functional differentiation of AHL family genes during plant evolution, which may be the result of the adaptive evolution of maize as a high-nitrogen-demand crop to cope with low nitrogen stress [34]. This unique expression pattern of *ZmAHL10* also suggests that it plays a specific and important regulatory role in maize response to low nitrogen stress, making it a core candidate gene for subsequent functional verification.

Sequence and subcellular localization analysis of *ZmAHL10* further clarified its molecular characteristics as a transcriptional regulator [41]. *ZmAHL10* has a full-length CDS of 1134 bp encoding 378 amino acids, and its protein is a hydrophilic protein with a molecular weight of about 37.19 kDa, which is consistent with the structural characteristics of the *ZmAHL* family. *ZmAHL10* contains two AT-hook domains at amino acid positions 97–109 and 155–167, which enables it to specifically bind to AT-rich DNA sequences and regulate gene expression. Secondary structure analysis showed that random coils account for 82.80% of *ZmAHL10*, which is consistent with the structural requirements of transcription factors for flexibility in DNA binding and protein interaction [41]. Subcellular localization experiments directly confirmed that *ZmAHL10* is localized in the nucleus, which further supports its functional localization as a transcriptional regulator that exerts biological functions by binding to nuclear DNA and regulating downstream gene expression.

Heterologous expression in Arabidopsis was used to verify the low nitrogen tolerance function of *ZmAHL10*, and the results showed that *ZmAHL10* can significantly enhance plant low nitrogen tolerance. Under normal nitrogen conditions, there were no significant differences in growth status between *ZmAHL10* overexpression lines, Arabidopsis ahl10 mutant lines and wild-type plants, indicating that *ZmAHL10* has no significant effect on plant normal growth. However, under low nitrogen stress, the ahl10 mutant lines showed significant growth retardation, while the *ZmAHL10* overexpression lines exhibited better growth status, with significantly higher shoot dry weight and total nitrogen content than the wild type. These results confirm that *ZmAHL10* can improve plant low nitrogen tolerance by enhancing nitrogen uptake capacity, which is consistent with the classical regulatory pathway of plants in response to low nitrogen stress—activating the expression of nitrogen transporter genes (e.g., NRT family) and nitrogen assimilation enzyme genes (e.g., NR and GS) to improve nitrogen uptake and utilization efficiency [42,43]. We speculate that *ZmAHL10* may directly or indirectly regulate the expression of these key nitrogen metabolism genes, thereby promoting nitrogen absorption and accumulation in plants under low nitrogen stress. In addition, photosynthetic pigment detection showed that under low nitrogen stress, the contents of chlorophyll a, chlorophyll b and carotenoids in *ZmAHL10* overexpression lines were significantly higher than those in wild-type and mutant lines, and the chlorophyll a/b ratio was significantly lower than that in the wild type. It is well known that the chlorophyll a/b ratio usually increases under low nitrogen stress due to the degradation of chlorophyll b [28], and the lower chlorophyll a/b ratio in *ZmAHL10* overexpression lines indicates that *ZmAHL10* can effectively alleviate the degradation of photosynthetic pigments caused by low nitrogen stress. This effect enables *ZmAHL10* overexpression lines to maintain the stability of the photosynthetic system under low nitrogen stress, ensure normal photosynthetic efficiency, and thus promote biomass accumulation [44,45]. Notably, *ZmAHL10* does not inhibit plant growth under normal nitrogen conditions, which is superior to many stress-responsive genes that cause growth retardation when overexpressed [46]. This characteristic makes *ZmAHL10* an ideal candidate gene for maize low nitrogen tolerance molecular breeding, which can improve maize low nitrogen tolerance without causing yield loss under normal nitrogen conditions, having important application value in agricultural production.

In conclusion, this study systematically identified the maize *ZmAHL* gene family and confirmed the low nitrogen tolerance function and preliminary mechanism of *ZmAHL10*. This research addresses a gap in understanding the maize AHL gene family’s response to low nitrogen stress and offers new gene resources and a theoretical foundation for molecular breeding aimed at enhancing low nitrogen tolerance in maize. Nevertheless, several limitations persist that warrant further investigation in future research. First, the regulatory role of *ZmAHL10* under combined drought and low nitrogen stress remains unexplored. In agricultural practice, crops frequently encounter multiple abiotic stresses simultaneously, with the interaction between drought and low nitrogen stress being particularly common [47]. Future studies should examine the expression patterns and regulatory mechanisms of *ZmAHL10* under these combined stresses, elucidating its synergistic role in plant responses and providing theoretical support for breeding maize varieties that can withstand multiple stresses. Second, the functional verification of *ZmAHL10* was mainly based on the heterologous expression system in Arabidopsis, and its function in the original species of maize needs to be further confirmed. Third, the direct downstream target genes of *ZmAHL10* involved in nitrogen uptake and the stability of the photosynthetic system remain unidentified. Future studies should employ ChIP-seq technology to screen for DNA sequences directly bound by *ZmAHL10*. Additionally, yeast one-hybrid and dual-luciferase reporter systems can be utilized to validate the regulatory relationship between *ZmAHL10* and its target genes. This approach aims to elucidate the core molecular pathway through which *ZmAHL10* regulates low nitrogen tolerance in maize, thereby enhancing our understanding of the molecular mechanisms underlying this process.

## 4. Conclusions

In summary, this study identified 35 *ZmAHL* genes in maize, and expression analysis revealed significant differences in the expression of this gene family in response to low nitrogen and drought stress. Notably, *ZmAHL10* exhibited pronounced characteristics associated with low nitrogen response, suggesting its potential regulatory role in maize adaptation to low nitrogen stress. Functional verification in a heterologous *Arabidopsis thaliana* system demonstrated that *ZmAHL10* significantly enhanced plant tolerance to low nitrogen; transgenic *Arabidopsis thaliana* lines overexpressing *ZmAHL10* displayed superior growth, increased nitrogen accumulation, and a more stable photosynthetic system under low nitrogen conditions. Mechanistically, this effect may be mediated by *ZmAHL10* through the enhancement of plant nitrogen absorption capacity and the alleviation of photosynthetic pigment degradation. These findings advance our understanding of the functional potential of the *ZmAHL* gene family in maize and underscore *ZmAHL10* as a promising candidate gene for further validation in studies on potato tolerance to low nitrogen. Future research should focus on overexpressing this gene in maize to confirm the direct role of *ZmAHL10* in low nitrogen tolerance. Additionally, investigating *ZmAHL10* as a downstream target gene regulated by transcription factors will provide a more robust molecular foundation for breeding low nitrogen-tolerant maize varieties.

## 5. Materials and Methods

### 5.1. Plant Materials and Low Nitrogen Treatment

We selected the maize inbred line B73, characterized by a well-defined genetic background and stable traits, along with the Arabidopsis ecotype Col-0 as experimental materials. The seeds were obtained from the Soil and Water Conservation Research Institute of Northwest A&F University. Following sterilization with 75% ethanol for 1 min and 100% ethanol for 30 s, Arabidopsis seeds were rinsed 5–6 times with sterile water and evenly sown on Hoagland solid medium. The seeds underwent vernalization at 4 °C for 48 h before being transferred to an artificial climate chamber for cultivation. The growth conditions were established as follows: light intensity of 120 μmol·m^−2^·s^−1^, a photoperiod of 16 h light and 8 h dark, a temperature maintained at 22 °C, and relative humidity sustained at 60–70%. After 10 days of growth, Arabidopsis seedlings were transplanted into seedling pots containing disinfected soil (vermiculite: perlite = 3:1, *v*/*v*) with specifications of a 7 cm upper mouth diameter, a 5 cm lower mouth diameter, and a height of 7 cm. Four plants were placed in each pot, with three replicates established (yielding a total of 12 plants per strain), and the plants continued to be cultivated in the aforementioned artificial climate chamber. Maize seeds were sterilized using the same method.

We utilized an enhanced Hoagland nutrient solution for irrigation, where the composition of the solid culture medium mirrors that of the nutrient solution employed for soil irrigation, with the addition of 1.2% (*w*/*v*) plant agar to ensure solidification. When ammonium nitrate (NH_4_NO_3_) serves as the sole nitrogen source, the complete concentrations of the nutrient solutions treated with two types of nitrogen are as follows: 2.0 mmol·L^−1^ CaCl_2_·2H_2_O, 0.75 mmol·L^−1^ K_2_SO_4_, 0.75 mmol·L^−1^ KCl, 0.65 mmol·L^−1^ MgSO_4_·7H_2_O, 0.25 mmol·L^−1^ KH_2_PO_4_, and 0.2 mmol·L^−1^ Fe EDTA. Additionally, the concentrations include 1 × 10^−3^ mmol·L^−1^ ZnSO_4_·7H_2_O, 1 × 10^−3^ mmol·L^−1^ MnSO_4_·H_2_O, 1 × 10^−4^ mmol·L^−1^ CuSO_4_·5H_2_O, 5 × 10^−6^ mmol·L^−1^ (NH_4_)_6_Mo_72_O_2_·24 × 4H_2_O, and 1 × 10^−3^ mmol·L^−1^ H_3_BO_3_. The normal nitrogen treatment (NN) employs an NH_4_NO_3_ concentration of 7.5 mmol·L^−1^, while the low nitrogen treatment (LN) utilizes an NH_4_NO_3_ concentration of 0.25 mmol·L^−1^.

Following sterilization, Arabidopsis seeds were uniformly inoculated on Hogland solid medium under both normal and low nitrogen conditions to facilitate germination and growth. For soil cultivation, Arabidopsis thaliana seedlings, which had germinated for 10 days and were of uniform age, were carefully transplanted into sterile soil seedling pots. Each pot received 10 mL of nutrient solution every three days to ensure consistent nutrition. Additionally, sterile deionized water was added as needed to maintain the substrate’s relative humidity between 60% and 70%. After 14 days of treatment, the growth of the seedlings was assessed.

### 5.2. Identification of AHL Gene Family in Maize

In order to identify the *AHL* gene in maize, the *AHL* gene was extracted from the maize genome database maizegdb (https://www.maizegdb.org/ (accessed on 21 October 2025)). We downloaded the protein sequence and annotation file of the whole genome of maize and used the reported AHL family conserved domain (AT-hook motif, PF02178; PPC/duf 296 domain (PF03479) as the search sequence; Hmmer 3.0 software was used to retrieve the hidden Markov model (e-value < 10^−5^), and preliminarily screened the candidate genes of the *AHL* family. Then, we used smart (http://smart.embl-heidelberg.de/ (accessed on 5 November 2025)). Online tools were used to verify the conserved domains of candidate genes, eliminate the genes without complete at-hook and PPC/duf296 domains, and finally determine the members of AHL gene family using ExPASY protparam (https://web.expasy.org/protparam/ (accessed on 5 November 2025)) Online tools were used to analyze the physical and chemical properties of AHL family members (molecular weight, isoelectric point, amino acid composition, etc.)

### 5.3. Structural Analysis of AHL Gene Family in Maize

Using TBtools-II (V2. 450) [48] software, the phylogenetic tree was constructed by maximum likelihood (ML) method, and bootstrap was set to 1000 repetitions. Using meme online software (https://meme-suite.org/meme/index.html (accessed on 7 November 2025)), we analyzed the conservative motif and set the maximum number of motifs to 10. Using batch CD search in the NCBI website (https://www.ncbi.nlm.nih.gov/Structure/bwrpsb/bwrpsb.cgi (accessed on 7 November 2025)), software was used to analyze the gene structure and conservative domain. The exon–intron structure of AHL family genes was identified by gene structure deployed in server online prediction software. According to the annotation file of maize genome, the gene density of 200 KB genetic interval and the position of *ZmAHL* gene on the chromosome were obtained. The density file was transformed into the gradient color heat map on the maize chromosome, and the TBtools [48] software was used for visualization.

### 5.4. Intraspecific and Interspecific Collinearity Analysis of AHL Gene in Maize

We downloaded the maize genome annotation file (gff3 format) and genome sequence from the maizegdb database and used the mcscanx plug-in of tbtools software to analyze the intraspecific collinearity of *ZmAHL* gene, set e-value < 1^−10^, screen the collinearity gene pairs, and draw the intraspecific collinearity map of maize chromosome *ZmAHL* gene. Interspecific collinearity analysis selected rice (model monocotyledonous plant) as the control, downloaded the whole genome annotation file and genome sequence of *Arabidopsis thaliana* from TAIR database, and used tbtools software to draw the visualization map of interspecific collinearity of *ZmAHL* gene between maize and rice.

### 5.5. Real Time Fluorescent Quantitative PCR (qPCR) Detection

The RNA-Seq data of different maize varieties under low nitrogen and drought were previously saved by the research group. In this study, using inbred line B73 maize, the gene expression pattern of *ZmAHL10* in leaves under low nitrogen (0.5 mmol · L^−1^) treatment for 0 d, 1 d, 3 d, 5 d, 7 d and 9 d was analyzed by quantitative real-time PCR (QRT PCR). The total RNA was extracted by Trizol reagent after grinding with liquid nitrogen. The integrity was detected by 1.2% agarose gel electrophoresis, and the purity was detected by nanodrop 2000 (Thermo Fisher Scientific, Waltham, MA, USA) (a260/A280 = 1.8~2.0). We took 1 μg of qualified total RNA, removed genomic DNA according to the instructions of the reverse transcription Kit (No. AE311, TransGen Biotech Co., Ltd., Beijing, China), and then conducted cDNA synthesis (20 μL system). The reaction conditions were 42 °C for 2 min, 37 °C for 15 min, and 85 °C for 5 s. The synthetic product was stored at −20 °C for standby. Using cDNA as a template, primer3 (https://www.primer3plus.com/ (accessed on 15 January 2025)) specific primers were designed by online software. The internal reference gene was act1 (attached Table S3). The expression of the target gene was detected by qPCR Kit (ChamQ Universal SYBR qPCR Master Mix; Cat. No. Q711; Vazyme Biotech Co., Ltd., Nanjing, China)). The reaction procedure was pre-denatured at 95 °C for 30 s, denatured at 95 °C for 5 s, and annealed at 60 °C for 30 s (40 cycles). The specificity of primer was verified by subsequent melting curve. Three biological and three technical replicates were set for each sample, and the relative expression was calculated by ^2^(−ΔΔCt) method.

### 5.6. Sequence Analysis of ahl10 Gene in Maize

Protscale was used to predict the hydrophilicity and hydrophobicity of protein using smart to predict protein domains (https://smart.embl.de/smart (accessed on 15 November 2025)). Protein secondary structure prediction was performed by online software sopma (https://npsa.lyon.inserm.fr/cgi-bin/npsa_automat.pl?page=/NPSA/npsa_sopma.html (accessed on on 15 November 2025)). Protein 3D structure prediction was performed through swissmodel online website (https://swissmodel.expasy.org/interactive (accessed on 15 November 2025)).

### 5.7. Subcellular Localization of ahl10 in Maize

Using the pcambia2300 vector preserved in our laboratory, which contains GFP tag, *ZmAHL10* was constructed on the vector by homologous recombination method and introduced into Agrobacterium (gv3101), and the Agrobacterium with GFP empty plasmid was used as the control group. The positive colonies with correct sequencing were cultured in Kans resistant LB liquid medium and collected. The cells were resuspended in MES buffer (10 mm MES, 10 mM MgCl_2_·6H_2_O, 200 μm acetyl eugenone), and the OD600 value was adjusted to 0.5. After the bacterial solution was left in the dark for 2–3 h, Agrobacterium tumefaciens was injected into tobacco leaves with a 1 mL syringe, and the transformed leaves were cultured at 24 °C for 2–3 days. The fluorescence signal of GFP was observed by laser confocal microscope (TCS SP8 Sr, Leika, Wetzlar, Germany).

### 5.8. Plant Shoot Total Nitrogen Content

We accurately weighed 0.2 g of dried *Arabidopsis thaliana* shoot samples and measured the total nitrogen concentration using the H_2_SO_4_-H_2_O_2_ method. The results were determined using a high-resolution digital chroma automatic analyzer (AA3, SEAL Company, Röttenbach, Germany) [49]. Data are presented as “mean ± standard deviation”. Each sample has three biological replicates.

### 5.9. Construction of Gene Overexpression Materials and Detection of Mutants

As in Section 5.7, the constructed *ZmAHL10* GFP overexpression vector was transferred into Agrobacterium tumefaciens gv3101, and the bacterial solution concentration was adjusted to OD600 = 0.8 for Arabidopsis infection. *Arabidopsis thaliana* was infected by inflorescence infection. After the seeds were mature, the seeds were collected and screened for resistance (kana) on MS medium. After 14 days of growth, normal plants were selected and transplanted into soil for culture. The leaves were cut to detect the expression level (Supplementary Figure S2), the positive plants were selected, the seeds were collected per plant, and the resistance screening was continued on MS medium until the T2 generation seeds were obtained.

The homozygous mutant of Arabidopsis ahl10 (at2g33620, salk_010945c) used in the experiment was purchased from Arashare (https://www.arashare.cn/index (accessed on 14 December 2024)). Col-0 wild type was used as control. On signal website (http://signal.salk.edu/tdnaprimers.2.html (accessed on 15 January 2025)), the LP, RP and BP primer sequences on T-DNA of ahl10 were queried. The homozygotes were identified by three-primer PCR method (TP—PCR). The rosette leaves of 7–10-day-old resistant seedlings were taken, and the genomic DNA was extracted by CTAB method. The primers of LP, RP and BP were designed, and the two groups of primers of lp + rp and bp + rp were used for PCR amplification, respectively. The PCR products were subjected to agarose gel electrophoresis. If the LP + RP group had no bands but the BP + RP group had bands, the plant was a homozygous mutant (Supplementary Figure S3).

### 5.10. Determination of Photosynthetic Pigments

Fresh leaves (0.1 g) were cut into pieces, then 20 mL of 80% acetone was added and extracted in the dark at room temperature for 24 h until the leaves faded. After centrifugation, the supernatant was taken and the absorbance at 663, 645 and 652 nm was measured (Epoch microplate reader, BioTek, Winooski, VT, USA). The content of photosynthetic pigment (mg/g FW) was calculated according to the photosynthetic pigment formula:

Chlorophyll a content (mg/g) = (12.7d663 − 2.69d645) × v/(1000 × w);

Chlorophyll b content (mg/g) = (22.9d645 − 4.68od663) × v/(1000 × w);

Carotenoids (mg/g) = 4.367 × od470 − 0.014 × chlorophyll a − 0.454 × chlorophyll b

### 5.11. Statistical Analysis

All the experimental data were expressed as “mean ± SD”. The graphpad prism 9 software was used for statistical analysis. Student’s t test of bilateral independent samples was used for analysis. The statistical significance standard was *p* < 0.05, indicating significant difference, and *p* < 0.01, indicating extremely significant difference.

## Data Availability

The original contributions presented in this study are included in the article/Supplementary Material. Further inquiries can be directed to the corresponding author.

## Supplementary Materials

The following supporting information can be downloaded at: https://www.mdpi.com/article/10.3390/plants15071062/s1. Supplementary Figure S1. Analysis of cis-regulatory elements of *ZmAHL10* promoter; Supplementary Figure S2. Expression level analysis of *ZmAHL10* transgenic *Arabidopsis thaliana*; Supplementary Figure S3. Identification of the *atahl10* mutant; Supplementary Table S1. Physicochemical properties and location prediction of *ZmAHL* gene family proteins; Supplementary Table S2. Expression level changes in *ZmAHL* gene family in RNA-Seq; Supplementary Table S3. Primer sequences used in this study.

## References

[1] Luo N., Meng Q., Feng P., Qu Z., Yu Y., Liu D.L., Müller C., Wang P. (2023). China can be self-sufficient in maize production by 2030 with optimal crop management. Nat. Commun..

[2] Hu B., Liu E., Zong R., Gao X., Fu X., Gao Y., Li Q., Li Q. (2024). Response of Water and Nitrogen Utilization Efficiency of Maize to Phased Drought and Nitrogen Application. Chin. Agric. Sci. Bull..

[3] Wang Y., Zhang Y.P., Zhu G.Y., Liao H.X., Hou W.F., Gao Q., Wang Y. (2024). Effects of localized nitrogen supply on growth and development, water and nitrogen utilization of maize seedlings under drought stress. Sci. Agric. Sin..

[4] Shen C., Ji Z., Jiao W., Zhang S., Huang Y., Qin Y., Huang M., Kang S., Mo Z., Jiang B. (2026). OsWRI1a coordinates systemic growth responses to nitrogen availability in rice. Science.

[5] Wang Q., Li S., Li J., Huang D. (2024). The Utilization and Roles of Nitrogen in Plants. Forests.

[6] Wang L.L., Ma F., He M.M. (2024). Research progress on the effects of light-nitrogen interaction on plant growth and development and nitrogen metabolism. J. Hangzhou Norm. Univ. (Nat. Sci. Ed.).

[7] Li T.Y., Yao L., Zhong Y.X., Wang Y., Li W.F., Xu Y., Li D., Liu R., Li B., Zhang W.F. (2025). Nitrogen Fertilizer Demand in China in the Context of Green Development. Acta Pedol. Sin..

[8] Zhang Z., Liu P., Liu H., Zhou T., Sun L. (2025). Environmental Impact and Mitigation Potential of Anthropogenic Active Nitrogen Loss in Agricultural Production and Consumption System in the Yangtze River Delta Region. Res. Environ. Sci..

[9] Li Y., Wang Q., Gao S., Wang X., He A., He P. (2025). Effects of Water–Nitrogen Coupling on Root Distribution and Yield of Summer Maize at Different Growth Stages. Plants.

[10] FAO (2022). The State of Food and Agriculture 2022: Leveraging Agriculture for Climate Action.

[11] Yan Y.L., Li J.Q., Xu Q.S., Wei Q.Q., Liu X.X., Chi C.X., Kong Y.L., Zhu L.F., Tian W.H., Zhang J.H. (2024). Physiological and molecular mechanisms of nitrogen regulating plant absorption and utilization of different nutrients. Plant Physiol. J..

[12] Chen W., Li J., Zhu H., Chen J., Yao Q. (2016). A review of the regulation of plant root system architecture by rhizosphere microorganisms. Acta Ecol. Sin..

[13] Zhang P., Zhang R.-R., Du S.-T. (2015). Research advances in nitrate uptake and transport in plants. J. Plant Nutr. Fertil..

[14] Zhang X., Lin J.X., Shan X.Y. (2016). Research progress on inorganic nitrogen transporters and their phosphorylation regulation in *Arabidopsis thaliana*. Acta Bot. Sin..

[15] Li J., Xu L., Zhao Y., Rui X., Shi J., Liu D. (2022). Nitrogen Metabolism Involved in Low Nitrogen Stress in Plants: A Review. Chin. Agric. Sci. Bull..

[16] Gao Y., Xu Z., Zhang L., Li S., Wang S., Yang H., Liu X., Zeng D., Liu Q., Qian Q. (2020). MYB61 is regulated by GRF4 and promotes nitrogen utilization and biomass production in rice. Nat. Commun..

[17] Tang W., Ye J., Yao X., Zhao P., Xuan W., Tian Y., Zhang Y., Xu S., An H., Chen G. (2019). Genome-wide associated study identifies NAC42-activated nitrate transporter conferring high nitrogen use efficiency in rice. Nat. Commun..

[18] Jiang M., Song Y., Yang R., Zheng C., Zheng Y., Zhang H., Li S., Tan Y., Huang J., Shu Q. (2023). Melatonin activates the OsbZIP79-OsABI5 module that orchestrates nitrogen and ROS homeostasis to alleviate nitrogen-limitation stress in rice. Plant Commun..

[19] Yang L., Fang S., Liu L., Zhao L., Chen W., Li X., Xu Z., Chen S., Wang H., Yu D. (2025). WRKY transcription factors:Hubs for regulating plant growth and stress responses. J. Integr. Plant Biol..

[20] Zhang W.-M., Cheng X.-Z., Fang D., Cao J. (2022). AT-HOOK MOTIF NUCLEAR LOCALIZED (AHL) proteins of ancient origin radiate new functions. Int. J. Biol. Macromol..

[21] Chen W.F., Li N., Chen L.F., Yuan W.L. (2024). Plant AT-hook transcription factors and their biological functions. Plant Physiol. J..

[22] Karami O., Rahimi A., Mak P., Horstman A., Boutilier K., Compier M., van der Zaal B., Offringa R. (2021). An Arabidopsis AT-hook motif nuclear protein mediates somatic embryogenesis and coinciding genome duplication. Nat. Commun..

[23] Shi X., Yang T., Ren M., Fu J., Bai J., Cui H. (2024). AT-hook motif nuclear localized transcription factors function redundantly in promoting root growth through modulation of redox homeostasis. Plant J..

[24] Wong M.M., Bhaskara G.B., Wen T.N., Lin W.D., Nguyen T.T., Chong G.L., Verslues P.E. (2019). Phosphoproteomics of Arabidopsis Highly ABA-Induced1 identifies AT-Hook-Like10 phosphorylation required for stress growth regulation. Proc. Natl. Acad. Sci. USA.

[25] Zhou L., Liu Z., Liu Y., Kong D., Li T., Yu S., Mei H., Xu X., Liu H., Chen L. (2016). A novel gene *OsAHL1* improves both drought avoidance and drought tolerance in rice. Sci. Rep..

[26] Peng C., Huo C., Xie W., Xiang Z., Huo S. (2024). Response of Different Maize Genotypes to Low Nitrogen Stress and Trait Selection of Low Nitrogen Tolerant Varieties. Chin. Agric. Sci. Bull..

[27] Wang R., Zhong Y., Han J., Huang L., Wang Y., Shi X., Li M., Zhuang Y., Ren W., Liu X. (2024). NIN-LIKE PROTEIN3.2 inhibits repressor Aux/IAA14 expression and enhances root biomass in maize seedlings under low nitrogen. Plant Cell.

[28] Kitajima K., Hogan K.P. (2003). Increases of chlorophyll a/b ratios during acclimation of tropical woody seedlings to nitrogen limitation and high light. Plant Cell Environ..

[29] Good A.G., Shrawat A.K., Muench D.G. (2004). Can less yield more? Is reducing nutrient input into the environment compatible with maintaining crop production?. Trends Plant Sci..

[30] Aravind L., Koonin E.V. (1998). The AT-hook motif: A small DNA-binding domain in search of a function. Trends Biochem. Sci..

[31] Zhao J., Favero D.S., Peng H., Neff M.M. (2013). *Arabidopsis thaliana* AHL family modulates hypocotyl growth redundantly by interacting with each other via the PPC/DUF296 domain. Proc. Natl. Acad. Sci. USA.

[32] Huo Q., Zhang Z., Zhang K., Wang Q., Zhang W., Ye X., Lyu Q., Galbraith D.W., Ma Z., Song R. (2025). Exploring maize transcriptional regulatory landscape through large-scale profiling of transcription factor binding sites. Mol. Plant.

[33] Zhang W.-M., Fang D., Cheng X.-Z., Cao J., Tan X.-L. (2021). Insights Into the Molecular Evolution of AT-Hook Motif Nuclear Localization Genes in Brassica napus. Front. Plant Sci..

[34] Hirel B., Le Gouis J., Ney B., Gallais A. (2007). The challenge of improving nitrogen use efficiency in crop plants: Towards a more central role for genetic variability and quantitative genetics within integrated approaches. J. Exp. Bot..

[35] Morisawa G., Han-Yama A., Moda I., Tamai A., Iwabuchi M., Meshi T. (2000). AHM1, a novel type of nuclear matrix-localized, MAR binding protein with a single AT hook and a J domain-homologous region. Plant Cell.

[36] Wang M., Chen B., Zhou W., Xie L., Wang L., Zhang Y., Zhang Q. (2021). Genome-wide identification and expression analysis of the AT-hook Motif Nuclear Localized gene family in soybean. BMC Genom..

[37] Kumar A., Singh S., Mishra A. (2023). Genome-wide identification and analyses of the AHL gene family in rice (Oryza sativa). 3 Biotech.

[38] Schnable P.S., Ware D., Fulton R.S., Stein J.C., Wei F., Pasternak S., Liang C., Zhang J., Fulton L., Graves T.A. (2009). The B73 maize genome: Complexity, diversity, and dynamics. Science.

[39] Yu T., Ma X., Zhang J., Cao S., Li W., Yang G., He C. (2025). Progress in Transcriptomics and Metabolomics in Plant Responses to Abiotic Stresses. Curr. Issues Mol. Biol..

[40] Yang Z., Cao Y., Shi Y., Qin F., Jiang C., Yang S. (2023). Genetic and Molecular Exploration of Maize Environmental Stress Resilience: Towards Sustainable Agriculture. Mol. Plant.

[41] Weidemüller P., Kholmatov M., Petsalaki E., Zaugg J.B. (2021). Transcription factors: Bridge between cell signaling and gene regulation. Proteomics.

[42] Ngaio C.S., Jacqueline M. (2016). Matthews, Mechanisms of DNA-binding specificity and functional gene regulation by transcription factors. Curr. Opin. Struct. Biol..

[43] Liu H., Gao X., Fan W., Fu X. (2025). Optimizing carbon and nitrogen metabolism in plants: From fundamental principles to practical applications. J. Integr. Plant Biol..

[44] Murata N., Takahashi S., Nishiyama Y., Allakhverdiev S.I. (2007). Photoinhibition of photosystem II under environmental stress. Biochim. Biophys. Acta Bioenerg..

[45] Evans J.R. (1989). Photosynthesis and nitrogen relationships in leaves of C3 plants. Oecologia.

[46] Kim H., Lee K., Hwang H., Bhatnagar N., Kim D.-Y., Yoon I.S., Byun M.-O., Kim S.T., Jung K.-H., Kim B.-G. (2014). Overexpression of PYL5 in rice enhances drought tolerance, inhibits growth, and modulates gene expression. J. Exp. Bot..

[47] Ma X., Wang W., Zhang J., Jiang Z., Xu C., Zhu W., Shi B., Yang W., Su H., Wang X. (2025). NRT1.1B acts as an abscisic acid receptor in integrating compound environmental cues for plants. Cell.

[48] Chen C.J., Chen H., Zhang Y., Thomas H.R., Frank M.H., He Y.H., Xia R. (2020). TBtools: An Integrative Toolkit Developed for Interactive Analyses of Big Biological Data. Mol. Plant.

[49] Liu H., Wang Z.H., Li F., Li K., Yang N., Yang Y., Huang D., Liang D., Zhao H., Mao H. (2014). Grain iron and zinc concentrations of wheat and their relationships to yield in major wheat production areas in China. Field Crops Res..

