# Supplementary material for: Heterologous Expression of ZmAHL10 Gene Enhances Low Nitrogen Tolerance in Transgenic Arabidopsis
Source: Plants (Basel). 2026 Mar 31;15(7):1062. doi: 10.3390/plants15071062 (PMC13074375; doi:10.3390/plants15071062)
Supplement: Supplementary file 1 [file plants-15-01062-s001.zip › Supplementary figures.pdf]

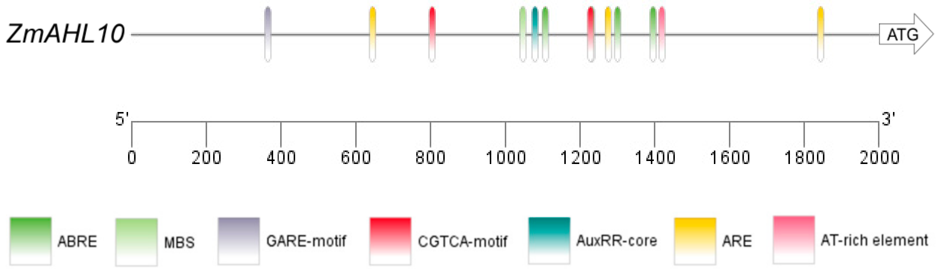

Figure S1 **Analysis of cis regulatory elements of *ZmAHL10* promoter** The distribution of predicted cis regulatory elements within the 2000bp upstream promoter region of the *ZmAHL10* gene, with a particular focus on elements related to environmental stress response.

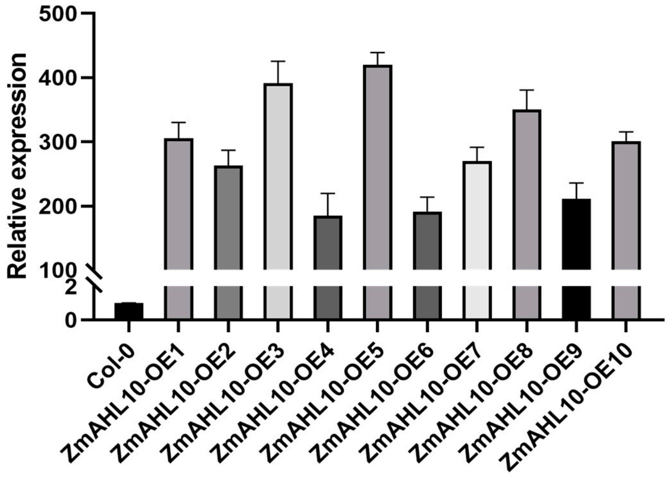

Figure S2 **Expression level of *ZmAHL10* in transgenic *Arabidopsis thaliana*** The expression level of *ZmAHL10* in transgenic lines and Col-0 *Arabidopsis thaliana*. *AtActin* serves as an internal reference gene. The bar chart represents the mean ± standard deviation (n=3 independent biological replicates).

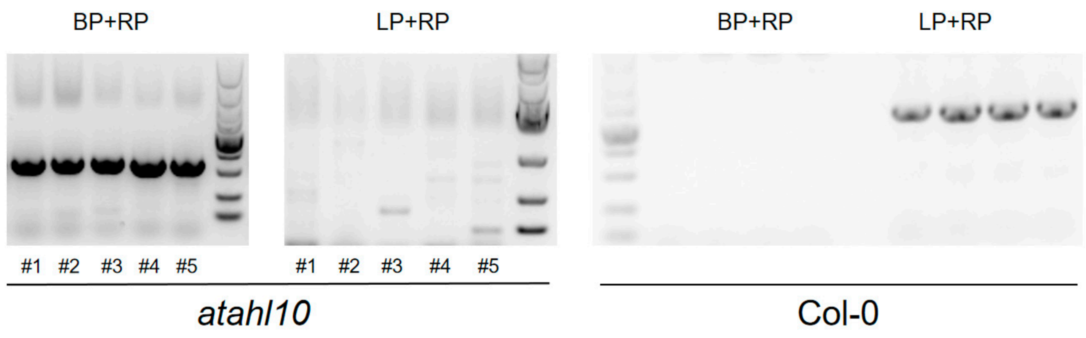

Figure S3 **Identification of the *atahl10* mutant** The agarose gel electrophoresis image for PCR identification of the *atahl10* mutant, with LP, RP, and BP as PCR identification primers.
